# Supplementary material for: Provider cost of treating oral potentially malignant disorders and oral cancer in Malaysian public hospitals
Source: PLoS One. 2021 May 13;16(5):e0251760. doi: 10.1371/journal.pone.0251760 (PMC8118562; doi:10.1371/journal.pone.0251760)
Supplement: S1 Table — (PDF) [file pone.0251760.s001.pdf]

S1 Table. Healthcare resource utilization frameset

Cost component	Elements	Unit	Utilization data source	Unit cost (MYR)	Cost data source
Outpatient	Specialist clinic visit	Frequency	Medical record	120.00	Fees (Medical)(Cost of Services) Order 2014
	Combined clinic	Frequency	Medical record	120.00	Fees (Medical)(Cost of Services) Order 2014
	Combined clinic (IKN) transport	Frequency	Medical record	141.96	ambulance MYR1.40/KM for 101.4 KM
	Medication	Packaging	Medical record	Based on the respective fee list	Hospital pharmacy price list of medicines
	Procedures	Each	Medical record	Based on the respective fee list	Fees (Medical)(Cost of Services) Order 2014
Biopsy	Incisional biopsy	Frequency	Medical record	1033.00	Fees (Medical)(Cost of Services) Order 2014
	FNAC	Frequency	Medical record	794.00	Fees (Medical)(Cost of Services) Order 2014
Investigation	Diagnostic/ laboratory tests	Each	Medical record	Based on the respective fee list	Fees (Medical)(Cost of Services) Order 2014
Inpatient (surgical)	Medical ward	Days	Medical record	160.00	Fees (Medical)(Cost of Services) Order 2014
	ICU Ward	Days	Medical record	360.00	Fees (Medical)(Cost of Services) Order 2014
	Operating theatre	Days	Medical record	250.00	MOH Full Paying Patient Tariff 2014
	Excisional biopsy	Frequency	Medical record	3452.00	Fees (Medical)(Cost of Services) Order 2014
	Surgical Procedures	Each	Medical record	Based on the respective fee list	Fees (Medical)(Cost of Services) Order 2014
	Manpower (surgeon/ doctor/ dental surgery assistant)	Frequency	Operating theatre list	Number of personnel	NA
		Hours	Assumed 8 hours for local/regional flap; 13 hours for free flap reconstruction; and 2 hours for OPMD, per person per session	58.43 (specialist)<br>43.45 (doctors)<br>15.30 (dental surgery assistant)	Salary list<br>(average salary divided by 22 days X 8 hours)
	Prostheses	Each	Medical record	Based on the respective fee list	Price quotation
	Medication	Packaging	Medical record	Based on the respective fee list	Hospital price list of medicines
	Support services	Each	Medical record	Based on the respective fee list	Fees (Medical)(Cost of Services) Order 2014
Inpatient (Oncology)	Radiotherapy treatment	Fractions	Medical record	500.00	Fees (Medical)(Cost of Services) Order 2014
	Radiotherapy ward	Days	Assumed from number of fractions (5 fractions=7 days)	160.00	Fees (Medical)(Cost of Services) Order 2014
	CT Simulation and computerized planning	Frequency	Assumed for each therapy (1 test per regimen)	5000.00	Fees (Medical)(Cost of Services) Order 2014
	Standard investigation	Regimen	Medical record	766.00	Fees (Medical)(Cost of Services) Order 2014
	Chemotherapy regimen	Cycle	Medical record	Calculated based on the treatment protocol	Fees (Medical)(Cost of Services) Order 2014 and Medicines Price List
	Chemotherapy ward	Days	Assumed from the cycle (1 cycle = 7 days)	160.00	Fees (Medical)(Cost of Services) Order 2014
