# Supplementary material for: Provider cost of treating oral potentially malignant disorders and oral cancer in Malaysian public hospitals
Source: PLoS One. 2021 May 13;16(5):e0251760. doi: 10.1371/journal.pone.0251760 (PMC8118562; doi:10.1371/journal.pone.0251760)
Supplement: S2 Table — (PDF) [file pone.0251760.s002.pdf]

## S2 Table. Inpatient factors impacting cost difference (MYR) between early- and late-stage cancer

| Factors[b] | Early-stage cancer | | Late-stage cancer | | *p-value*[a] |
|---|---|---|---|---|---|
| | *Mean* | *SD* | *Mean* | *SD* | |
| Chemotherapy | 4,384 | 3,621 | 5,992 | 5,328 | 0.6182 |
| Radiotherapy | 27,762 | 2,680 | 28,878 | 8,682 | 0.0620 |
| Surgery procedure cost | 21,526 | 7,331 | 26,978 | 12,316 | 0.0010 |
| Reconstructive surgery cost | 6,313 | 4,642 | 8,330 | 4,575 | 0.0051 |
| Human resource cost | 2,219 | 1,357 | 3,454 | 3,187 | 0.0024 |
| Hospitalization cost | 3,057 | 1,377 | 3,813 | 3,092 | 0.3821 |

[a] Kruskal-Wallis H test with significance set to $p<0.05$
