# Supplementary material for: Provider cost of treating oral potentially malignant disorders and oral cancer in Malaysian public hospitals
Source: PLoS One. 2021 May 13;16(5):e0251760. doi: 10.1371/journal.pone.0251760 (PMC8118562; doi:10.1371/journal.pone.0251760)
Supplement: S3 Table — (PDF) [file pone.0251760.s003.pdf]

S3 Table. Annual cost estimate and difference based on IPW method (MYR)[a]

<table>
<tr><th rowspan="2"></th><th colspan="2">OPMD</th><th colspan="2">I</th><th colspan="2">II</th><th colspan="2">III</th><th colspan="2">IV</th></tr>
<tr><th>Mean</th><th>SD</th><th>Mean</th><th>SD</th><th>Mean</th><th>SD</th><th>Mean</th><th>SD</th><th>Mean</th><th>SD</th></tr>
<tr><td>Initial phase</td><td>5,705</td><td>6,655</td><td>60,382</td><td>19,856</td><td>46,099</td><td>16,892</td><td>64,803</td><td>22,778</td><td>74,405</td><td>27,730</td></tr>
<tr><td>Mean difference (SE) between stages</td><td colspan="2" rowspan="2">-</td><td colspan="4">-14,283 (5,813)
95% CI [-25,678; -2,889], p= 0.037</td><td colspan="4">9,602 (5,195)
95% CI [-581; 19,784], p= 0.065</td></tr>
<tr><td>Mean difference (SE) between Early and Late-stage</td><td colspan="8">18,862 (4,022)
95% CI [10,979; 26,746], p< 0.001</td></tr>
<tr><td>Mean difference (SE) between OPMD and cancer group</td><td colspan="10">58,818 (2,330)
95% CI [54,252; 63,385], p< 0.001</td></tr>
<tr><td>Maintenance</td><td>1,383</td><td>1,555</td><td>4,364</td><td>8,545</td><td>1,477</td><td>735</td><td>3,606</td><td>3,532</td><td>4,266</td><td>6,573</td></tr>
<tr><td>Mean difference (SE) between stages</td><td colspan="2" rowspan="2">-</td><td colspan="4">-2,887 (2,618)
95% CI [-8,017; 2,244], p= 0.270</td><td colspan="4">660 (1,548)
95% CI [-2,374; 3,694], p= 0.670</td></tr>
<tr><td>Mean difference (SE) between Early and Late-stage</td><td colspan="8">1,120 (1,504)
95% CI [-1,829; 4,070], p= 0.457</td></tr>
<tr><td>Mean difference (SE) between OPMD and cancer group</td><td colspan="10">2,152 (733)
95% CI [717; 3,589], p= 0.003</td></tr>
</table>

[a] Annual cost based on the inverse probability weighting method by Bang and Tsiatis
