# Supplementary material for: Provider cost of treating oral potentially malignant disorders and oral cancer in Malaysian public hospitals
Source: PLoS One. 2021 May 13;16(5):e0251760. doi: 10.1371/journal.pone.0251760 (PMC8118562; doi:10.1371/journal.pone.0251760)
Supplement: S4 Table — (PDF) [file pone.0251760.s004.pdf]

S4 Table. Literature review of oral/oropharyngeal cancer management cost worldwide

Author	Methodology	Resource	Costing	Duration	Cost, USD[a]
Lang et al., 2004 [US]	Retrospective cohort analysis	Medicare reimbursement	Healthcare	5 years	58,737 (in-situ)<br>66,996 (localized)<br>84,324 (distant)<br>91,614 (regional)
Epstein et al., 2007 [US]	Retrospective, Observational	Insurance claims database	Healthcare	1 year [b]	32,212 (early)<br>39,316 (late)
Dedhia et al., 2011 [US]	Retrospective, Observational	Medicare reimbursement, published reports, estimation	Healthcare	1 year [b]	13,189 (early)<br>70,381 (late)
Jacobson et al., 2012 [US]	Retrospective, Observational	Commercial insurance/ claim database	Healthcare, indirect cost	1 year [b]	94,359
Lairson et al., 2017 [US]	Retrospective Observational	commercial insurance claims database	Healthcare	2 years	142,042
Agthoven et al., 2001 [Netherland]	Retrospective, micro-costing method	Financial data, worktime estimation	Direct cost	2 years	48,191 (primary)<br>60,914 (recurrent)
Speight et al., 2006 [UK]	Retrospective, ABC	case record abstraction, reference cost	Healthcare	1 year [b]	16,225 (early)<br>21,747 (late)
Kim et al., 2011 [UK]	Retrospective, ABC	database record abstraction, reference cost	Healthcare	1 year [b]	34,793
Pollaers et al., 2019 [Australia]	Retrospective	case record abstraction, finance record	Healthcare	1 year [b]	54,561 (early)<br>76,709 (late)
Zavras et al., 2002 [Greece]	Retrospective cohort analysis, ABC	case record abstraction, treatment tariff, reference values	Healthcare	3 years	6,354 (early)<br>14,456 (late)
Han et al., 2010 [China]	Retrospective cohort analysis, ABC	case record abstraction, treatment tariff	Healthcare	1 year [b]	6,318 (early)<br>9,751 (late)
Rezapour et al., 2018 [Iran]	Retrospective, decision-analytic model	case record abstraction, expert opinion, telephone interviews, treatment tariff	Healthcare, productivity	1 year [b]	2,261(early)<br>10,704 (late)
Amarasinghe, et al., 2019 [Sri Lanka]	Retrospective, ABC	case record abstraction, interview	Healthcare, patient cost	1 year [b]	412 (SII)<br>2,118 (late)

* *ABC*= activity-based costing

[a] cost inflated to 2019 using Consumer Price Index (CPI) and converted to USD using purchase power parity (PPP)

[b] first-year cost
