# Supplementary material for: Provider cost of treating oral potentially malignant disorders and oral cancer in Malaysian public hospitals
Source: PLoS One. 2021 May 13;16(5):e0251760. doi: 10.1371/journal.pone.0251760 (PMC8118562; doi:10.1371/journal.pone.0251760)
Supplement: Attachment — Submitted filename: Review #1-MR.pdf [file pone.0251760.s005.pdf]

Summary

The article attempts to determine the costs of treating oral potentially malignant disorders (OPMD) and oral cancer in Malaysia from a healthcare provider's perspective. The authors have aimed to fill an important missing gap in the current literature on the cost of OPMD and oral cancer in Malaysia and also to provide policy recommendation on the need for early detection and prevention.

Besides trying to fill an important gap in literature, the study includes both East and West Malaysia resources use which is one of the key strengths of the study as well as the comprehensiveness of the study.

However, the study has several limitations which may need to be addressed:

Time of study period is short (6 months):

- To do a comprehensive costing, time-period should be longer to capture follow-up treatment of cancer and resources use. Explain why this time period is used and whether the study could be extended for a longer time period.

Background

- Provide background of health system in Malaysia and whether all the costs determined in the analysis are covered by the government. Are there any out-of-pocket costs or costs from private insurers that are not included?

Costing seems incomplete.

- Since the study did not fully include one of the key drivers of costs based on external institutions data (hospitalization and utilization of chemo) and instead relied on expert opinion (perhaps one 1 expert), the costing is not fully accurate. To do a full costing, attempts should be made to get data from the National Cancer Institute and other external institutes to obtain the resource use of hospitalizations and chemo treatment.
- Provide more details on the constitution of the inter-disciplinary team – which institutes etc.? Any reason a hospital pharmacist was not involved who might be able to provide more cost details and treatment pathways?
- More information required on the types of chemo drugs that were considered as part of the costing.
- Also needs to show the variation of costs for different patients– may need some form of probability distribution function to show the variation in costs is recommended.
- Consider inclusion of process map that shows the journey of the patient through the healthcare system
- The title only indicates oral cancer when the study includes both oral cancer and OPMD costing.

Method

- More rationale provided on the IPW method and why the authors think this costing method is the most appropriate for undertaking this study and how the costs were adjusted for follow-up

and survival. Have the authors considered other methods and if so, why are those methods excluded from the study? How does the study handle uncertainty in costs and the skewed nature of healthcare costs?

- What constitute maintenance costs?
- The study did not seem to include any administrative costs in the total costs (e.g. utilities, personnel, surgical overhead costs). These may need to be determined or approximated so that costs provided are not under-estimated.
- Inclusion of sensitivity analysis to show variation in costs

Minor Edit

- Suggest to change the word ‘manpower’ to personnel, workforce or professional services etc.
